# Ultrasound‐guided embolization for renal artery pseudoaneurysm in solitary kidney

**DOI:** 10.1002/iju5.12796

**Published:** 2024-10-22

**Authors:** Sana Augustine, Mitwa Patel, Pugazhendi Inban, Sk Sadia Rahman Synthia, Ummul Z Asfeen, Aliza Yaqub, Aadil Mahmood Khan, Mansi Singh

**Affiliations:** ^1^ Liaquat University of Medical and Health Sciences Hyderabad Pakistan; ^2^ David Tvildiani Medical University Tbilisi Georgia; ^3^ Government Medical College Omandurar Chennai India; ^4^ M.H. Samorita Medical College and Hospital Dhaka Bangladesh; ^5^ New York Medical College at St Michael's Medical Center Newark New Jersey USA; ^6^ Bahria University Medical and Dental College Karachi Pakistan; ^7^ OSF Healthcare System University of Illinois College of Medicine Peoria Illinois USA; ^8^ Bogomolets National Medical University Kyiv Ukraine

**Keywords:** partial nephrectomy, percutaneous nephrolithotomy (PCNL), renal artery pseudoaneurysm (RAP), solitary kidney, ultrasound‐guided embolization

## Abstract

**Introduction:**

Renal artery pseudoaneurysm is a rare yet serious complication following percutaneous nephrolithotomy, especially in patients with solitary kidneys. Effective management is crucial to prevent further renal damage.

**Case presentation:**

We report a case of a 41‐year‐old male with a solitary kidney who experienced gross hematuria and renal insufficiency 3 months after percutaneous nephrolithotomy. Due to the patient's renal insufficiency and the risks associated with arterial catheterization, ultrasound‐guided embolization was chosen as the treatment approach. Initial angiographic attempts were impeded by renal vessel spasms, delaying intervention. However, successful direct percutaneous embolization was subsequently performed using ultrasound and digital subtraction angiography.

**Conclusion:**

The patient's recovery was uneventful, and follow‐up assessments showed no recurrence of renal artery pseudoaneurysm. This case highlights the effectiveness of ultrasound‐guided embolization as a viable treatment option for post‐percutaneous nephrolithotomy renal artery pseudoaneurysm, particularly in patients with solitary kidneys.

**Abbreviations & Acronyms**
PCNL: percutaneous nephrolithotomy
RAP: renal artery pseudoaneurysm

**Keynote message**

This case report showcases the successful use of ultrasound‐guided embolization for managing renal artery pseudoaneurysm in patients with solitary kidneys. By highlighting the efficacy and safety of this approach, it offers valuable insights for clinicians facing similar challenges, emphasizing the importance of preserving renal function while effectively treating vascular complications.

## Introduction

Hemorrhage resulting from RAP is an uncommon yet significant complication that may arise following renal trauma, biopsy, percutaneous nephrostomy, PCNL, and partial nephrectomy. Although the occurrence of this potentially life‐threatening complication is below 1%, its incidence is expected to rise due to the growing adoption of endoscopic renal procedures.^1^ However, the risk of RAP is higher in the case of PCNL done in a solitary kidney because of the hypertrophy of the renal parenchyma.^2^ Renal angiography can be used for diagnosing RAP of an interlobar artery. Ultrasound guidance presents a distinctive alternative to fluoroscopy for percutaneous renal access. In addition to avoiding ionizing radiation exposure for both the patient and intraoperative staff, it provides numerous benefits, such as improved visualization of the posterior renal calyx and adjacent visceral structures.^1^, ^3^ We present a 41‐year‐old male with a solitary right kidney presented with hematuria and a RAP post‐PCNL and its comprehensive management.

## Case history

A 41‐year‐old male with a solitary right kidney presented with hematuria and episodic fever 3 months after PCNL. He had a percutaneous nephrostomy tube in place with daily hemorrhagic fluid output of 200–300 mL and reduced urine output. The tube was retained to ensure adequate kidney drainage, monitor for residual fragments or complications, and manage delayed healing of the nephrostomy tract. The patient had received 13 units of blood transfusion post‐PCNL. No other significant medical history was reported.

### Examination

The patient appeared fatigued with stable vital signs and right flank tenderness. The percutaneous nephrostomy tube site was clean. Laboratory results showed hemoglobin of 8.4 g/dL, serum creatinine of 3.2 mg/dL, and a urine creatinine ratio of 22.9 mg/dL.

### Investigations

**Fig. 1:**
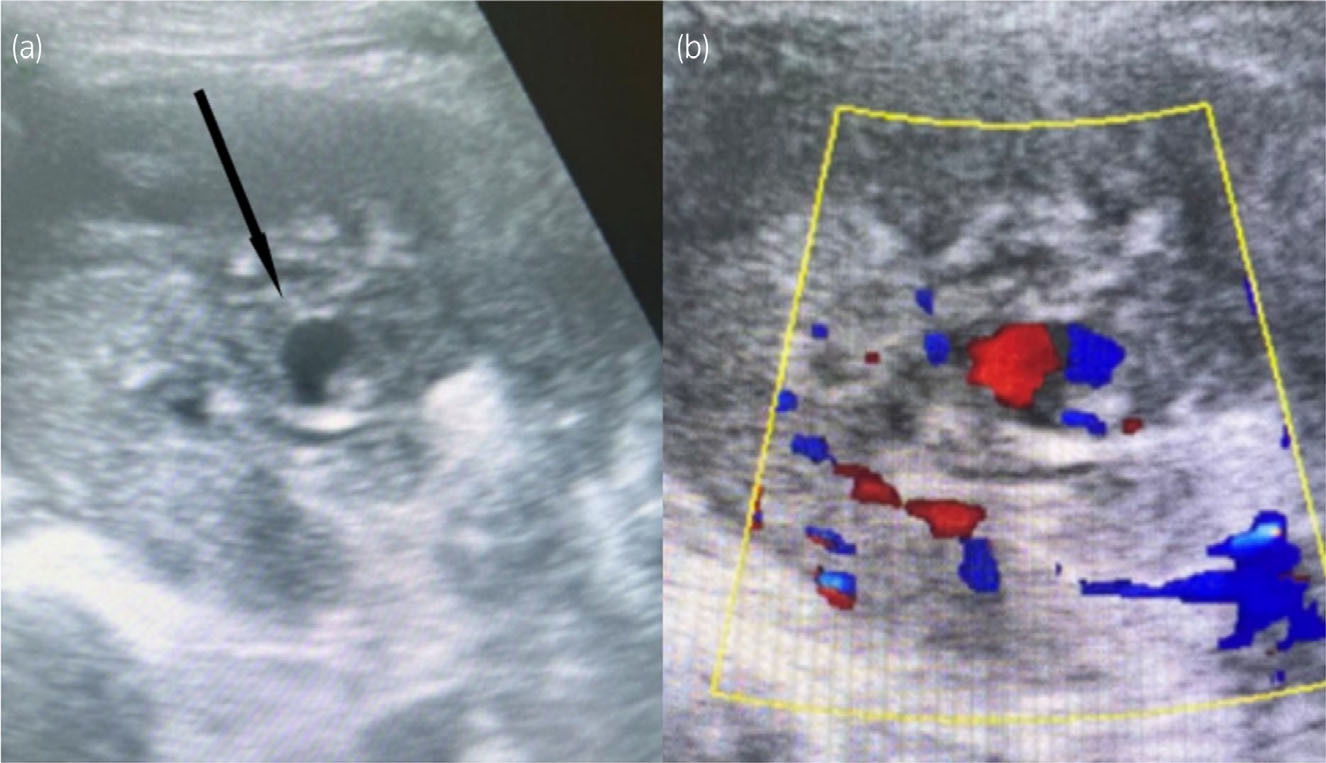
B‐mode ultrasonography (a) reveals an anechoic cystic lesion at the midpole of the kidney, while the accompanying Color Doppler image (b) demonstrates turbulent flow within the lesion, indicative of a renal artery pseudoaneurysm.

The radiological imaging and ultrasonography revealed hypertrophied right kidney and perinephric collections of 100 mL. Additionally, the presence of a PCN tube was noted. Most importantly, an anechoic cystic lesion at the midpole of the kidney was identified. Later, ultrasound Doppler imaging confirmed that there was turbulent flow within this anechoic lesion, strongly suggesting that there was a RAP, as shown in Figure 1. These findings collectively guided the clinical evaluation and treatment approach for the patient's hematuria and related symptoms.

### Treatment and results

**Fig. 2:**
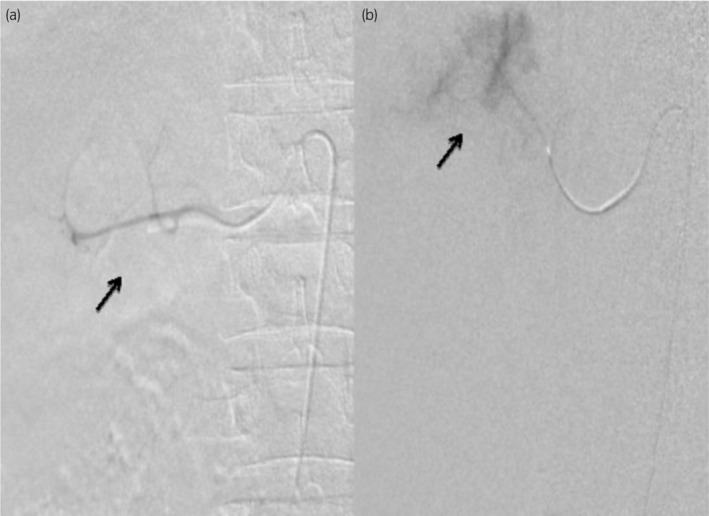
Digital subtraction angiography images showcasing the right renal artery at both midpole (a) and upper‐pole (b) regions, performed using a 5F SIMS catheter.

After consulting with the interventional radiology team, the management of this complex case aimed to address a RAP following a recent PCNL procedure. A 5F vascular sheath and 5F SIMS catheter were used to access the right femoral artery, allowing catheter advancement to the right renal artery for angiography, as shown in Figure 2. However, complications arose as renal vessels entered spasm, preventing further catheter progression. Despite administering intravascular vasodilators, the arterial spasm persisted, necessitating the procedure's abandonment due to the heightened risk of arterial dissection.

**Fig. 3:**
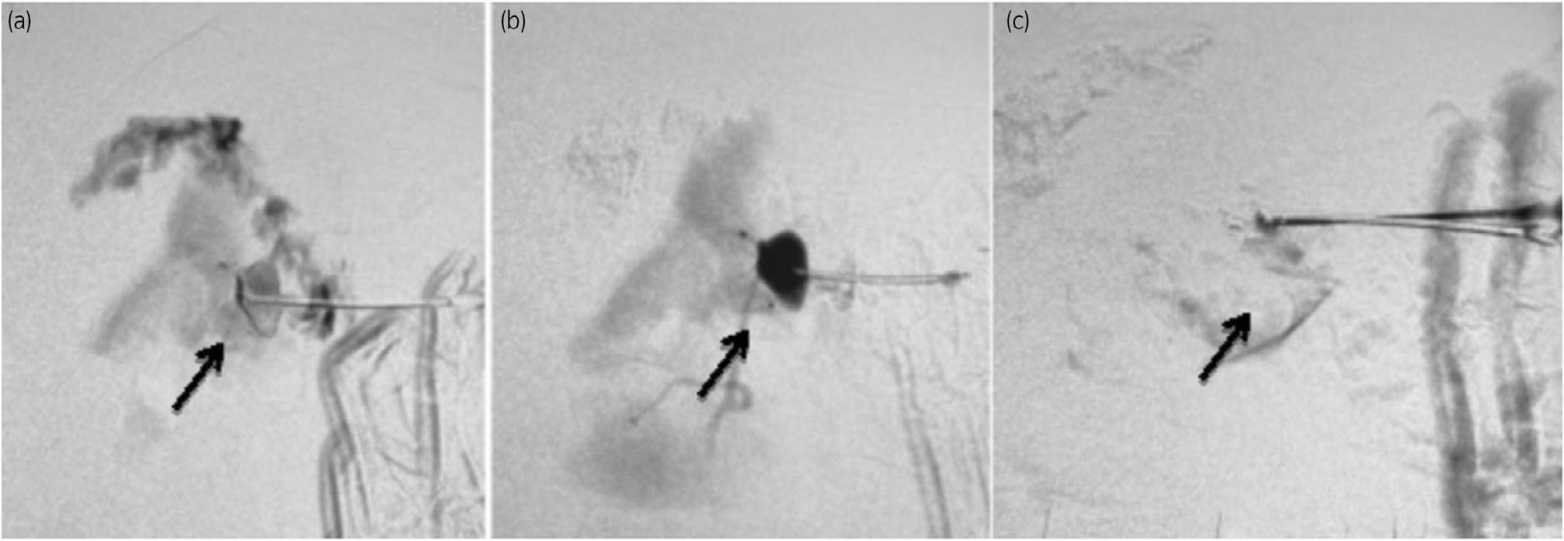
Simultaneous percutaneous puncture of the pseudoaneurysm under sonographic guidance viewed concurrently in digital subtraction angiography (DSA) (a), followed by DSA depiction of the contrast‐filled pseudoaneurysm (b), and, finally, DSA showing the absence of opacification in the pseudoaneurysm following N‐butyl cyanoacrylate glue injection (c).

A more successful approach was pursued, where a direct percutaneous embolization procedure was meticulously planned under the guidance of ultrasound and digital subtraction angiography (DSA). This involved the percutaneous puncture of the RAP using an 18‐G vygon needle, guided by ultrasonographic imaging. An angiogram, conducted under DSA guidance with the use of a water‐soluble radiographic contrast agent (Visipaque), confirmed the presence of the contrast‐filled pseudoaneurysm. Subsequently, 0.1 mL of 1:2 N‐butyl cyanoacrylate glue, reconstituted with lipiodol, was slowly and precisely injected into the pseudoaneurysm under DSA guidance, with careful fluoroscopy monitoring as shown in Figure 3. Remarkably, this approach minimized the utilization of Visipaque contrast, thereby reducing radiation exposure compared to conventional computed tomography or angiography. Post‐procedure vitals remained stable, and there were no discernible complications observed in the postoperative period.

### Follow‐up

A follow‐up color Doppler ultrasound 24 h post‐procedure showed no flow in the previously problematic pseudoaneurysm, confirming the successful resolution of the RAP and validating the effectiveness of the management approach.

## Discussion

PCNL is the preferred method for removing kidney stones but can occasionally lead to complications such as renal arteriovenous fistulas or pseudoaneurysms, which are typically asymptomatic or present with temporary symptoms.^4^ RAP is a rare but recognized complication following PCNL, where an artery is partially severed or punctured, causing blood to leak into a confined hematoma.^5^ This complication often involves a significant arterial branch, such as a third‐order branch of the renal artery, which may be difficult to detect during the procedure due to thrombosis or spasm.^6^ Over time, dislodgement of the occluding clot can result in delayed hematuria. In this case report, we describe a patient with RAP in a solitary kidney who presented with hematuria and decreased urine output following PCNL.

Delayed bleeding after significant percutaneous procedures, often from arteriovenous fistulas or arterial pseudoaneurysms, can be managed effectively with selective angioembolization. Continuous bleeding usually points to an arteriovenous fistula, while intermittent bleeding suggests an arterial pseudoaneurysm. Both conditions are treated similarly with angioembolization, which has high success rates. Hospital admission and angiography are necessary for any post‐procedure bright red urine, as angiography is diagnostic in over 90% of cases.^7^

Direct ultrasound‐guided percutaneous embolization has emerged as a novel method for treating renal pseudoaneurysms. The percutaneous embolization technique involves the following steps:

- First, under local anesthesia and imaging guidance (typically fluoroscopy), a catheter is introduced into the vascular system, usually through the femoral artery.
- The catheter is then navigated to the target vessel, supplying the area of interest.
- Embolic agents, which can include coils, particles, or liquid embolics, are carefully introduced through the catheter to occlude the target vessel.
- The progress and effectiveness of the embolization are monitored in real‐time using angiographic imaging.
- Once the desired level of occlusion is achieved, the catheter is withdrawn, and hemostasis is achieved at the entry site.

This approach eliminates the need for contrast media, reduces radiation exposure hazards, and minimizes complications associated with angiographic catheterization. Notably, it also reduces the risk of surgical intervention, such as partial or total nephrectomy, which is particularly important in patients with solitary kidneys.^8^

Numerous studies and case reports have investigated the utility of ultrasound‐guided embolization for treating RAP in solitary kidneys post‐PCNL.^9^, ^10^, ^11^ For instance, Shah *et al*. documented a successful coil embolization case in a young female with RAP after PCNL.^1^ Additionally, various studies have examined different aspects of ultrasound‐guided techniques in PCNL procedures. Usawachintachit and Tzou emphasized the benefits of ultrasound guidance, such as real‐time imaging and reduced radiation exposure, during renal access and tract dilation in PCNL.^3^ These cases collectively demonstrate the effectiveness of ultrasound‐guided embolization for RAP in solitary kidneys. While percutaneous embolization presents several advantages, including minimally invasive nature and precise targeting of the affected vessels, it also carries potential risks. These include non‐target embolization, post‐embolization syndrome, and complications such as infection, bleeding, or vessel injury. Despite these risks, the benefits often outweigh the disadvantages, particularly in cases where surgical options pose higher risks. Moreover, the research suggests that ultrasound‐guided PCNL is a safe and feasible procedure with a low complication rate in patients with solitary kidneys. Long‐term follow‐up data revealed that over 90% of these patients experienced either improvement or stabilization of renal function after undergoing ultrasound‐guided PCNL.^12^

Diagnosis and management of RAP in patients with solitary kidneys pose significant challenges. Percutaneous ultrasound‐guided embolization should be considered, especially for patients with solitary kidneys and post‐PCNL hematuria. We report a case where a patient with a solitary kidney and severe hematuria required multiple blood transfusions. Due to renal insufficiency, pre‐operative CT angiography was not feasible, making ultrasound the only available guidance method. The procedure initially failed due to renal vessel spasms during selective catheterization but was ultimately managed with a minimally invasive super‐selective embolization technique. Percutaneous embolization is particularly advantageous in scenarios involving a single kidney due to the critical need to preserve renal function. However, it is also effective in a wide range of cases, providing a valuable treatment option for patients with vascular abnormalities in various organs. The case was successfully managed with ultrasoun‐ and fluoroscopic‐guided direct injection of cyanoacrylate glue into the pseudoaneurysm. Herein, we also discuss the unanticipated events during the embolization of the pseudoaneurysm in the solitary kidney and its management.

## Conclusion

In conclusion, our case report demonstrates the effective management of a RAP in a solitary kidney using ultrasound‐guided embolization. This non‐surgical approach successfully controlled bleeding and preserved renal function, highlighting the value of angioembolization in high‐risk patients. Ultrasound guidance proved to be a safe and precise method, minimizing complications and optimizing outcomes. Further research is needed to confirm the efficacy and safety of this technique in similar cases.

## Author contributions

Sana Augustine: Writing – original draft. Mitwa Patel: Writing – original draft. Pugazhendi Inban: Data curation; formal analysis. Sk Sadia Rahman Synthia: Supervision; validation. Ummul Z. Asfeen: Investigation; methodology. Aliza Yaqub: Methodology; visualization. Aadil Mahmood Khan: Writing – review and editing. Mansi Singh: Writing – review and editing.

## Conflict of interest

The authors declare no conflict of interest.

## Approval of the research protocol by an Institutional Reviewer Board

Not applicable.

## Informed consent

Written informed consent was obtained from the for publication of this case report and any accompanying images. A copy of the written consent is available for review by the editor in chief of this journal.

## Registry and the Registration No. of the study/trial

Not applicable.

## Data Availability

The datasets used and/or analyzed during the current study are available from the corresponding author on reasonable request.
